# Supplementary material for: The Effect of Intermittent versus Continuous Non-Invasive Blood Pressure Monitoring on the Detection of Intraoperative Hypotension, a Sub-Study
Source: J Clin Med. 2022 Jul 14;11(14):4083. doi: 10.3390/jcm11144083 (PMC9321987; doi:10.3390/jcm11144083)
Supplement: Supplementary file 1 [file jcm-11-04083-s001.zip › jcm-1771328-supplementary.pdf]

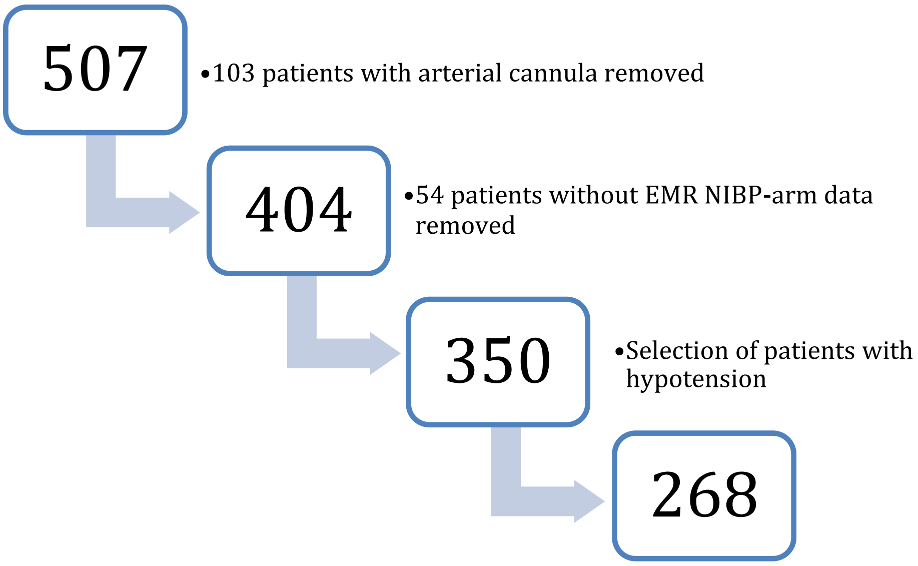

Supplementary Figure S1. Flowchart selection patients. The original study had 507 patients [15]. The first step was the selection of patients with non-invasive blood pressure monitoring. The second step was the removal of patients with missing NIBP-arm data. The third step was the selection of patients with at least one cNIBP-finger hypotensive event. Hypotension was defined as a MAP < 65 mmHg for at least one minute.

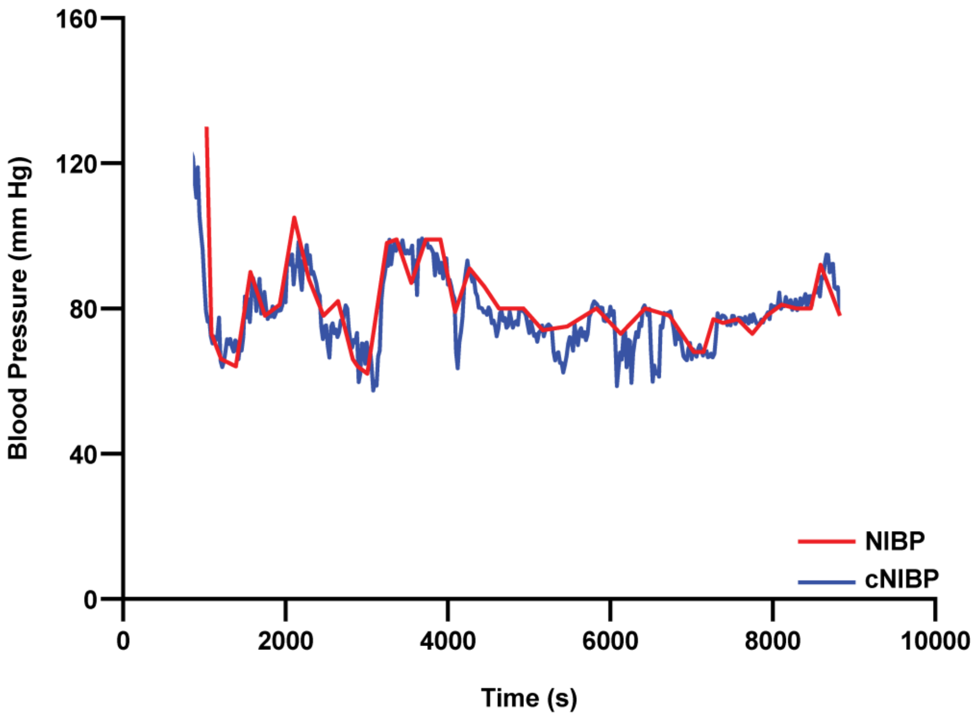

Supplementary Figure S2: Example of time synchronization of continuous (cNIBP-finger) and intermittent (NIBP-arm) blood pressure monitoring. Blue line = cNIBP-finger. Red line = NIBP-arm.

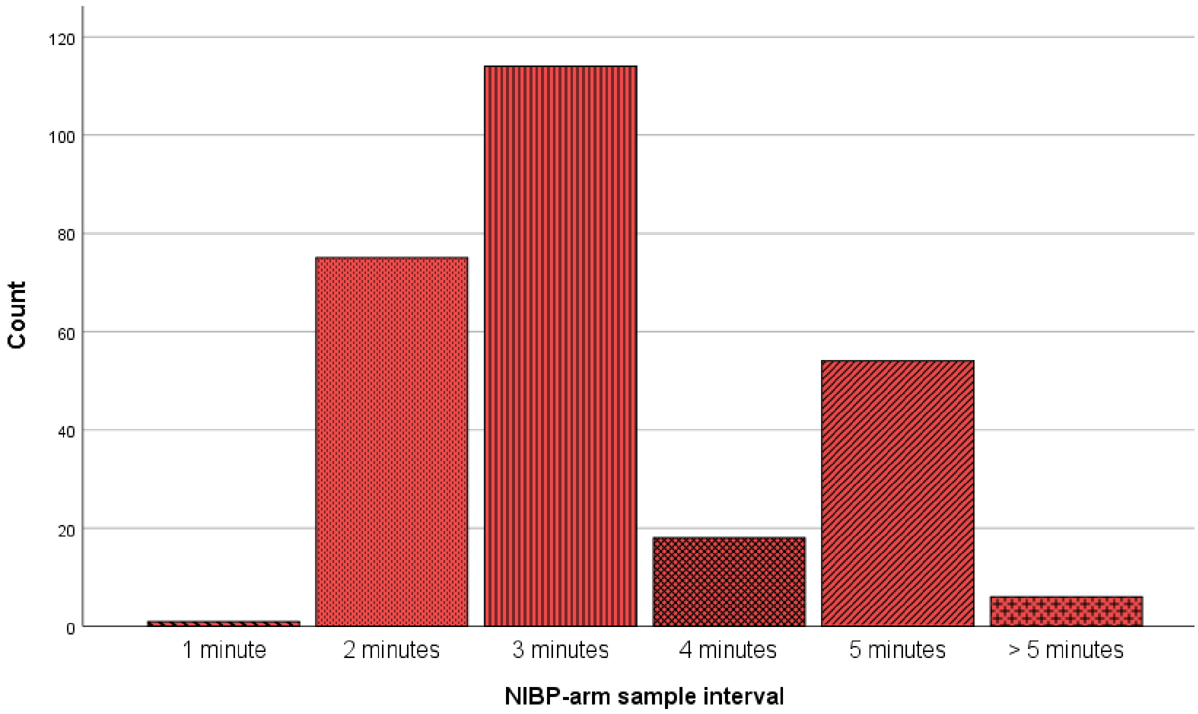

Supplementary Figure S3: Number of patients per NIBP-arm sample interval. The majority of anesthesiologists set the NIBP-arm interval for the majority of the anesthesia time at 3 minutes (*n* = 114, 43%), followed by 2 minutes (*n* = 75, 28%) and 5 minutes (*n* = 54, 20%).

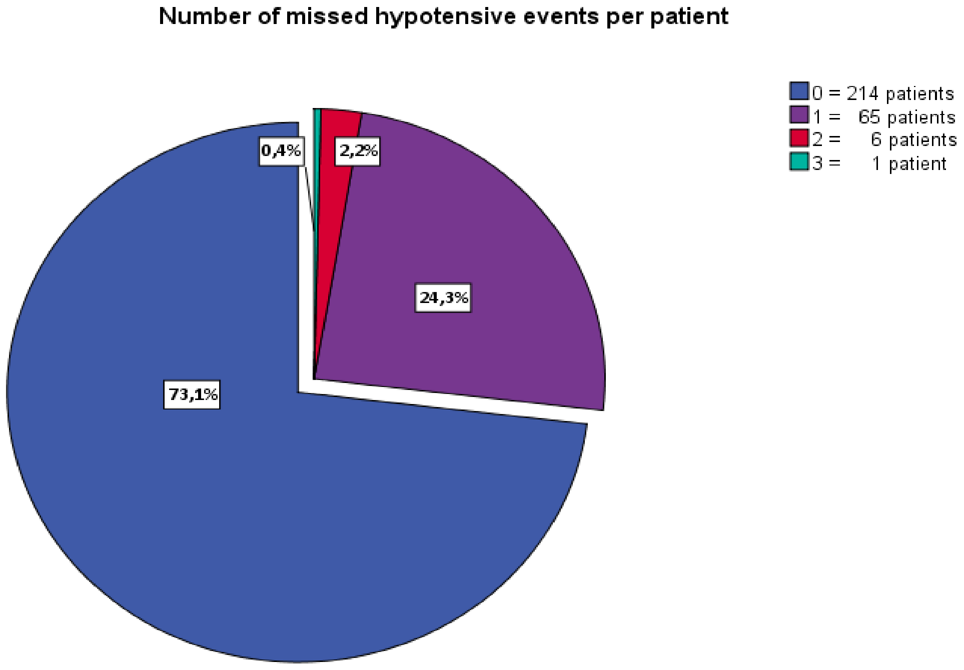

Supplementary Figure S4: Number of missed hypotensive events per patient.
